# Structure and function of an unusual R452-dependent monoclonal antibody against SARS-CoV-2

**DOI:** 10.1128/jvi.01844-24

**Published:** 2025-04-08

**Authors:** Bing Zhou, Qi Gui, Congcong Liu, Huimin Guo, Haiyan Wang, Lin Cheng, Qing Fan, Xiangyang Ge, Zheng Zhang, Bin Ju

**Affiliations:** 1 Institute for Hepatology, National Clinical Research Center for Infectious Disease, Shenzhen Third People’s Hospital, The Second Affiliated Hospital, School of Medicine, Southern University of Science and Technology 535206 , Shenzhen, Guangdong, China; 2 Department of Infectious Diseases, Affiliated Hospital of Southwest Medical University 556508 , Luzhou, Sichuan, China; 3 Guangdong Key Laboratory for Anti-infection Drug Quality Evaluation, Shenzhen, Guangdong, China; 4 Shenzhen Research Center for Communicable Disease Diagnosis and Treatment, Chinese Academy of Medical Sciences, Shenzhen, Guangdong, China; Loyola University Chicago - Health Sciences Campus, Maywood, Illinois, USA

**Keywords:** SARS-CoV-2 variant, R452-dependent neutralizing antibody, structural basis, strict restriction

## Abstract

The coronavirus disease 2019 (COVID-19) pandemic caused by severe acute respiratory syndrome coronavirus 2 (SARS-CoV-2) variants is still a major public health concern worldwide. Currently, SARS-CoV-2 variants have been widely used to develop the updated vaccine. However, whether these mutated residues still have good immunogenicity remains elusive. In particular, we know little about what kind of antibodies can be induced by the infection or vaccination of SARS-CoV-2 variants and their biological characteristics. Here, we identified an R452-dependent monoclonal neutralizing antibody, ConD-852, from a primarily Delta variant-infected individual, indicating that the mutated R452 residue has good immunogenicity. We determined the high-resolution cryo-electron microscopy (cryo-EM) structure of ConD-852 complexed with the Delta receptor-binding domain (RBD), revealing how it binds to the R452-related epitopes and their detailed interactions. Interestingly, ConD-852 could only bind to the amino acid residue “R” at the 452 position on RBD, displaying a strict restriction to recognize SARS-CoV-2. Overall, our findings regarding ConD-852 confirmed the good immunogenicity of SARS-CoV-2 variants carrying the L452R mutation and enriched our knowledge of the binding model involving the neutralizing antibody and the mutated virus.

**IMPORTANCE:**

Although SARS-CoV-2 variants have been widely used to update the COVID-19 vaccine candidate, whether these mutations still have good immunogenicity is unknown. This study demonstrates that the mutated R452 residue can induce potent neutralizing antibodies and reports a high-resolution cryo-EM structure of an R452-dependent monoclonal antibody binding to the epitopes around the R452 residue on SARS-CoV-2 RBD.

## INTRODUCTION

Since the end of 2020, severe acute respiratory syndrome coronavirus 2 (SARS-CoV-2) variants keep emerging and lead to a series of the coronavirus disease 2019 (COVID-19) waves, such as Alpha, Beta, Gamma, Delta, and various Omicron subvariants in order of time, including BA.1, BA.2, BA.4/5, BQ.1, XBB.1.5, BA.2.86/JN.1, KP.3, and LB.1 (1–10). Currently, Omicron subvariants are prevalent in many countries, posing a serious threat to human lives and global economy (10). It has been proven that the Omicron subvariants have the greatest reduction in the neutralizing capacity so far (7, 8, 11, 12), which largely reduces the neutralization elicited by the former wild-type (WT) and variant infections (13–17). Next-generation vaccines have been designed based on SARS-CoV-2 variant viruses or sequences, which are expected to fight against emerging variants (18–20). We must first determine whether the mutations in the variants still have good immunogenicity and if these mutations can induce neutralizing antibodies (nAbs), which is very important to design the updated variant vaccines. Biological samples acquired from primary exposure to SARS-CoV-2 variants are crucial for answering this question. During the Omicron pandemic, many reports were primarily based on the polyclonal antibody level of plasma or sera and monoclonal antibodies (mAbs) induced by breakthrough infection with SARS-CoV-2 variants (21–25). Newly emerging SARS-CoV-2 variants can not only escape the preexisting immunity but also elicit the variant-specific antibody response different from that induced by the prior strains (26). However, there are a few reports on the characteristics and function of these specific mAbs induced by primary infection of variants. Beta variant primary infection has been shown to induce effective antibody response targeting K417N, E484K, or N501Y mutated residues on the receptor-binding domain (RBD) of the spike protein (27). Before the emergence of Omicron, the Delta variant was the dominant one worldwide, which comprised more than 90% for quite a long time (28). A large number of people initially suffered from infection with the Delta variant, which provided us an opportunity to investigate the immunogenicity of some important mutated residues, especially those located in the RBD of spike proteins, such as L452R and T478K. Therapeutic antibodies are effective in preventing severe disease from SARS-CoV-2 infection and constitute an important option in pandemic preparedness, but mutations within the spike protein of variants confer resistance to many antibodies (29). The L452R mutation on the RBD of the spike protein of SARS-CoV-2 is a critical determinant for antibody escape (30, 31), having a significant influence on the interaction between the virus and nAbs. The changed amino acid at position 452 from leucine (a hydrophobic residue) to arginine (a hydrophilic and positively charged residue) might disrupt the binding interface between the spike protein and certain nAbs, particularly those relying on hydrophobic interactions or specific charge interactions with the original leucine residue (32). To understand the antigenic landscape of SARS-CoV-2 variants, we retrospectively studied the blood samples of Delta-infected donors stored in the biological sample bank (BioBank) of Shenzhen Third People’s Hospital and attempted to isolate and characterize the mutated residue-dependent monoclonal nAbs. Here, we report the identification of an R452-dependent mAb and reveal its structural basis and molecular mechanism for recognizing epitopes around the R452 residue.

## RESULTS

### Monoclonal nAbs isolated from a primarily Delta-infected individual

**Fig 1:**
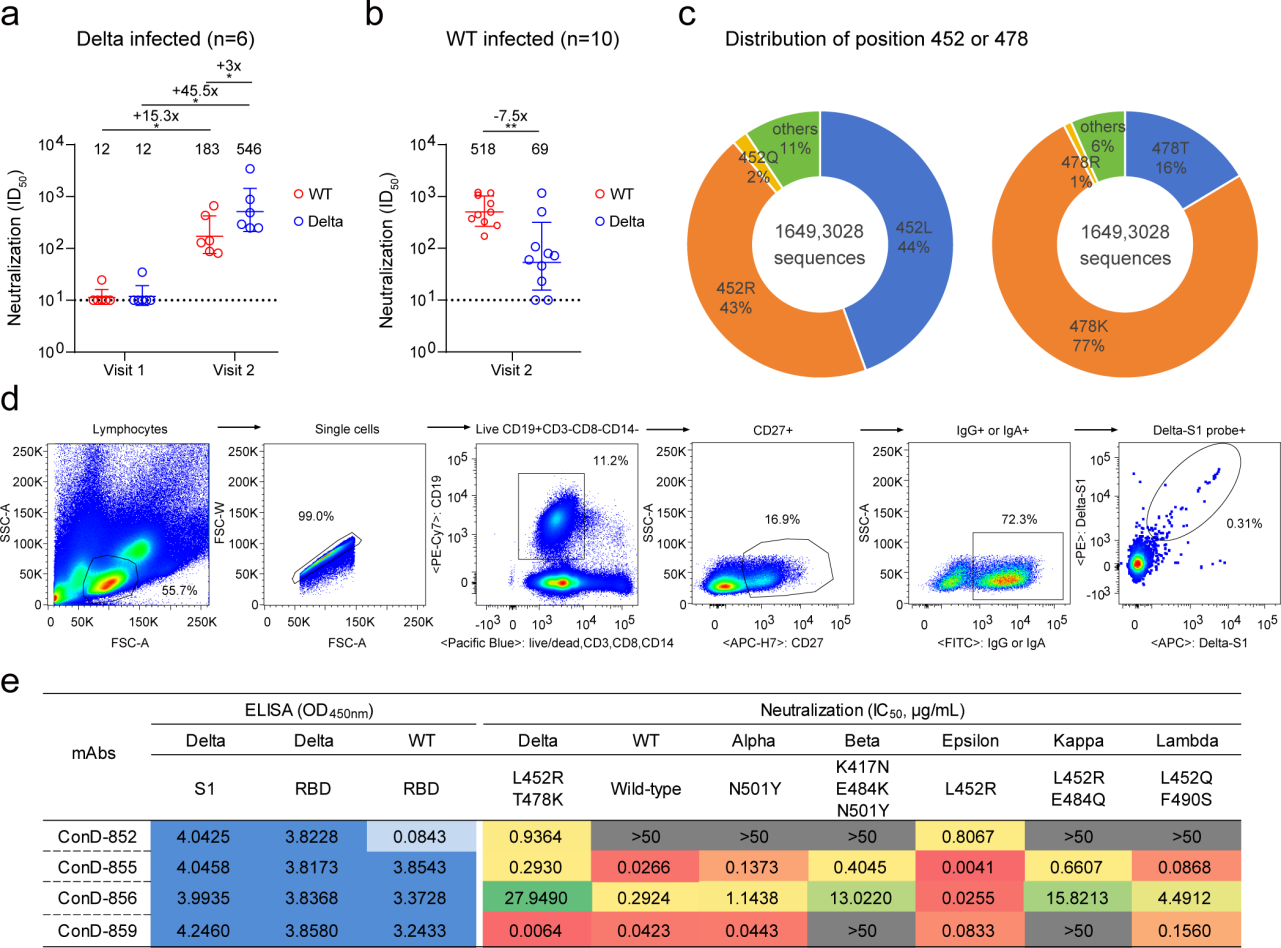
Biochemical characterization of antibodies derived from COVID-19 convalescent individuals infected with SARS-CoV-2 Delta variants. Plasma samples (Visit 1: early infection, Visit 2: clinical recovery) from individuals infected with (a) Delta variant and (b) WT SARS-CoV-2 were analyzed for their neutralization against pseudoviruses bearing the envelope glycoprotein of WT and Delta. (c) The overall distribution of spike protein at positions 452 and 478 among all sequences in GISAID. (d) Single-cell sorting of Delta-S1-specific memory B cells by flow cytometry. The gating strategy was CD19+CD3−CD8−CD14−CD27+IgG/IgA+S1+. (e) The binding and neutralizing activities of ConD-852, ConD-855, ConD-856, and ConD-859 against SARS-CoV-2 variants. Data in panels a, b, and e are means from two independent experiments. Data in panels a and b are presented in geometric mean values ± standard deviations. Statistical significance was determined using the two-tailed paired Wilcoxon test for mean values using the GraphPad Prism version 8 software. *P < 0.05, **P < 0.01.

In this study, we collected blood samples from six patients infected with the Delta variant in the early and recovery stages, all of whom were not immunized with any SARS-CoV-2 vaccine. Approximately 2 days after admission, none of the plasma samples displayed effective neutralization against Delta. The 50% inhibitory dilution (ID_50_) was lower than the limit of detection (1:20). During the recovery of all patients, their plasma samples exhibited high neutralizing activity (Fig. 1a; Fig. S1). The geometric mean ID_50_ against the Delta variant was significantly higher than that against the WT variant, suggesting that natural infection with Delta induced a strain-specific neutralizing antibody response. Contrastingly, the plasma of 10 convalescent patients recovered from the first WT SARS-CoV-2 infection showed more potent neutralization against WT SARS-CoV-2 than that against the Delta variant (Fig. 1b; Fig. S1). The SARS-CoV-2 Delta variant harbors two featured mutations in the RBD, L452R and T478K, which mediate its evasion from some WT viral infection-induced RBD-specific nAbs and vaccine immunization (5, 33, 34). Based on the sequences available in GISAID as of 24 September 2024, the frequencies of L452R and T478K were approximately 43% and 77%, respectively (Fig. 1c). These two substitutions have also been detected in many other SARS-CoV-2 variants, including CH.1.1, BA.4/5, BQ.1.1, EG.5.1.6, and JN.1 (12, 34–36). Therefore, we further investigated whether R452 or K478 could induce nAbs to determine the immunogenicity associated with these mutations at the mAb level.

We performed the isolation of mAbs from a convalescent individual infected with Delta (Donor 2) and tried to characterize mutation-dependent monoclonal nAbs. We used the S1 protein of the Delta variant as bait to sort Delta S1-specific single-memory B cells using flow cytometry (Fig. 1d). We successfully identified four monoclonal nAbs against the Delta variant using the methods we previously reported (34, 37–40) (Fig. 1e). After enzyme-linked immunosorbent assay (ELISA) screening for their specificity to the Delta S1 domain, ConD-852, ConD-855, ConD-856, and ConD-859 (all IgG subtypes) were detected to bind to the Delta RBD and WT RBD proteins, three of which exhibited cross-reactivity. ConD-852 did not react with the WT RBD, suggesting that it was a Delta RBD-specific monoclonal nAb. The results of the neutralization assay further reflected that ConD-852 should be an R452-dependent nAb because it only neutralized Delta harboring L452R and T478K mutations in the RBD and Epsilon carrying L452R yet could not inhibit WT SARS-CoV-2 infection. In contrast, ConD-855, ConD-856, and ConD-859 were broad nAbs cross-reacting with different SARS-CoV-2 variants such as Alpha, Beta, and Kappa.

### ConD-852 is an R452-dependent monoclonal nAb

**Fig 2:**
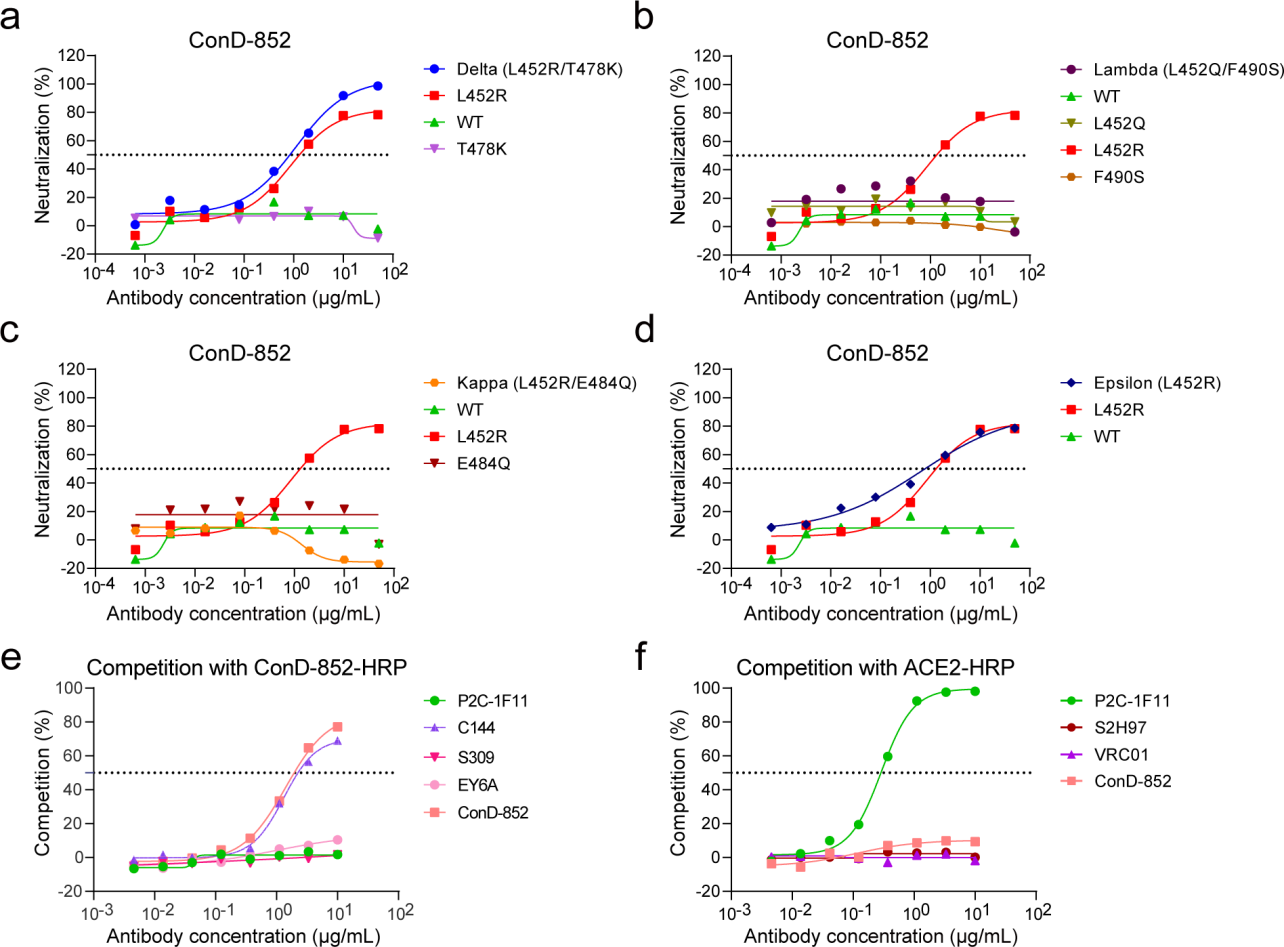
Epitope analysis of ConD-852 binding to Delta-RBD. Neutralization of ConD-852 against (a) Delta, L452R, WT, T478K; (b) Lambda, WT, L452Q, L452R, F490S; (c) Kappa, WT, L452R, E484Q; and (d) Epsilon, L452R, WT SARS-CoV-2 pseudoviruses. Competition ELISA of ConD-852 with (e) representative mAbs and (f) ACE2. The experiment was independently performed at least twice, and one representative result is shown.

Notably, the Kappa (L452R/E484Q) and Lambda (L452Q/F490S) variants were resistant to the neutralization of ConD-852, which is probably attributed to three other mutated residues (Q at 452, Q at 484, or S at 490). To further confirm the pivotal role of R452 in determining the interaction between the Delta RBD and ConD-852, we performed a deeper analysis of the influence of L452Q, T478K, E484Q, and F490S mutations. T or K at the 478 residue did not affect the neutralization of ConD-852 (Fig. 2a). Both L and Q at the 452 residue caused ConD-852 to lose its neutralization (Fig. 2b). Although ConD-852 recognized the R452 residue in the Kappa RBD, E484Q substitution largely affected its neutralizing activity (Fig. 2c), further narrowing the range of epitopes recognized by ConD-852. The single L452R mutation assay provided the most solid evidence for ConD-852 directly binding around R452 in the RBD (Fig. 2d). The competition ELISA revealed that ConD-852 bound to epitopes overlapping with C144 (41) (a Class 2 mAb, competing with ACE2 and binding to the RBD in both “up” and “down” conformations) but did not compete with ACE2, P2C-1F11 (37) (a Class 1 mAb, strongly competing with ACE2 yet only binding to the up RBD), S309 (42) (a Class 3 mAb, not competing with ACE2 and binding to the outer face of RBD), EY6A (43) (a Class 4 mAb, not competing with ACE2 and binding to the inner face of RBD), or S2H97 (44) (a Class 5 mAb, whose epitope is far away from the ACE2-binding site) (Fig. 2e and f). Overall, we predicted that ConD-852 was an R452-dependent mAb belonging to Class 2 nAbs, which was elicited by the SARS-CoV-2 Delta variant initial infection.

### Binding affinity of ConD-852

**Fig 3:**
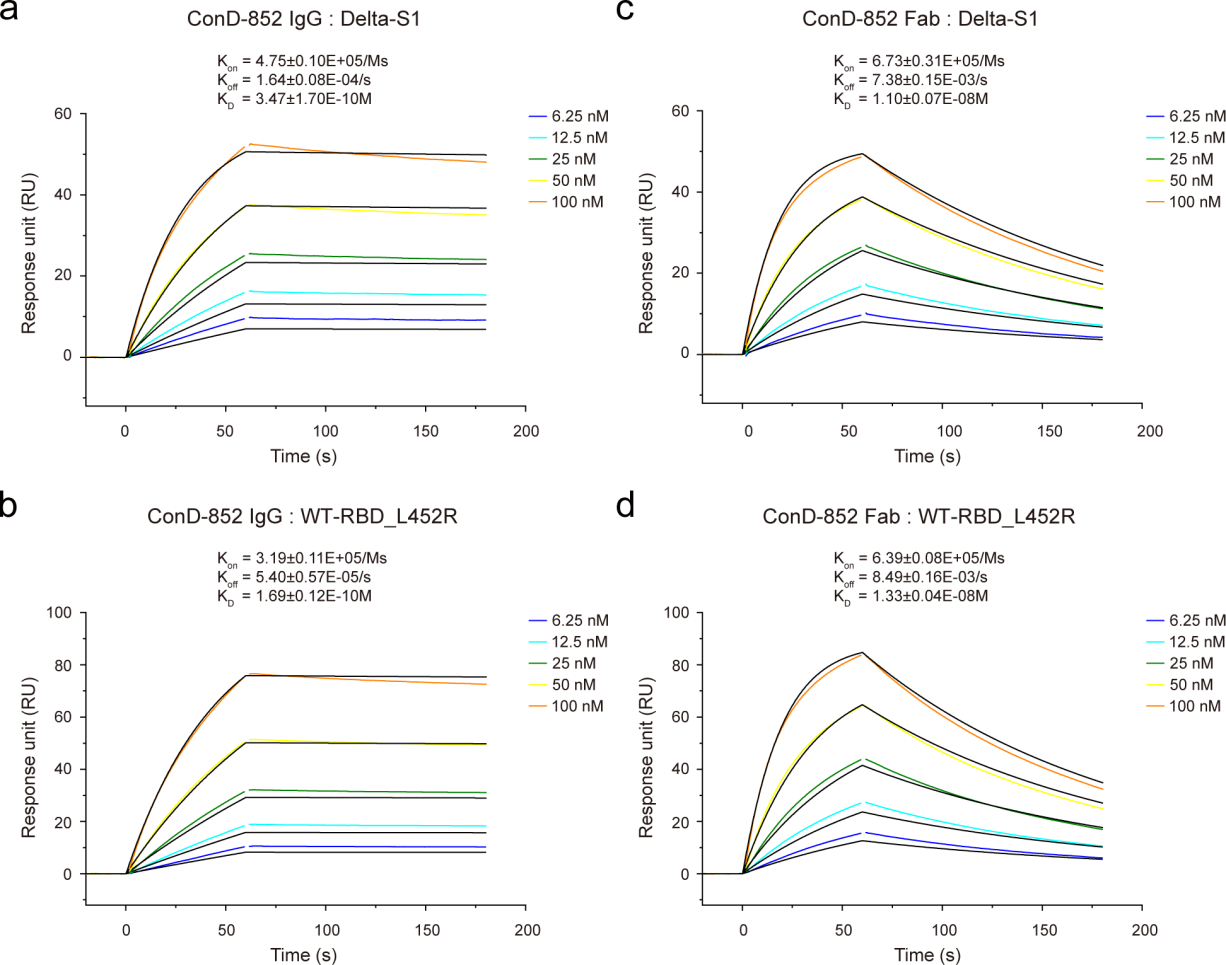
Binding affinity of IgG-form and Fab-form ConD-852 to Delta-S1 and WT-RBD_L452R proteins. SPR analysis of IgG-form ConD-852 binding to (a) Delta-S1 and (b) WT-RBD_L452R proteins. SPR analysis of Fab-form ConD-852 binding to (c) Delta-S1 and (d) WT-RBD_L452R proteins. The dissociation constant (*K*_D_), association rate constant (*K*_on_), and dissociation rate constant (*K*_off_) were calculated from two independent experiments and represented in mean values ± standard deviations. One representative curve is presented here.

The surface plasmon resonance (SPR) analysis revealed the binding affinity of ConD-852 to SARS-CoV-2. Delta-S1 protein and WT-RBD_L452R protein were immobilized to the SPR sensor chip and determined their binding kinetics for ConD-852, respectively. As shown in Fig. 3a and b, the IgG form of ConD-852 bound to Delta-S1 and WT-RBD _L452R with high affinities, whose dissociation constants were 0.35 and 0.17 nM, respectively. We also prepared the fragment antigen binding (Fab) of ConD-852 to further explore its binding capacity. Despite some decline, the Fab form of ConD-852 maintained a certain binding affinity to Delta-S1 (11 nM) and WT-RBD_L452R (13 nM) (Fig. 3c and d). The relatively high binding affinity of ConD-852 could support further research, such as structure determination.

### Structural basis of ConD-852 binding to delta RBD

**Fig 4:**
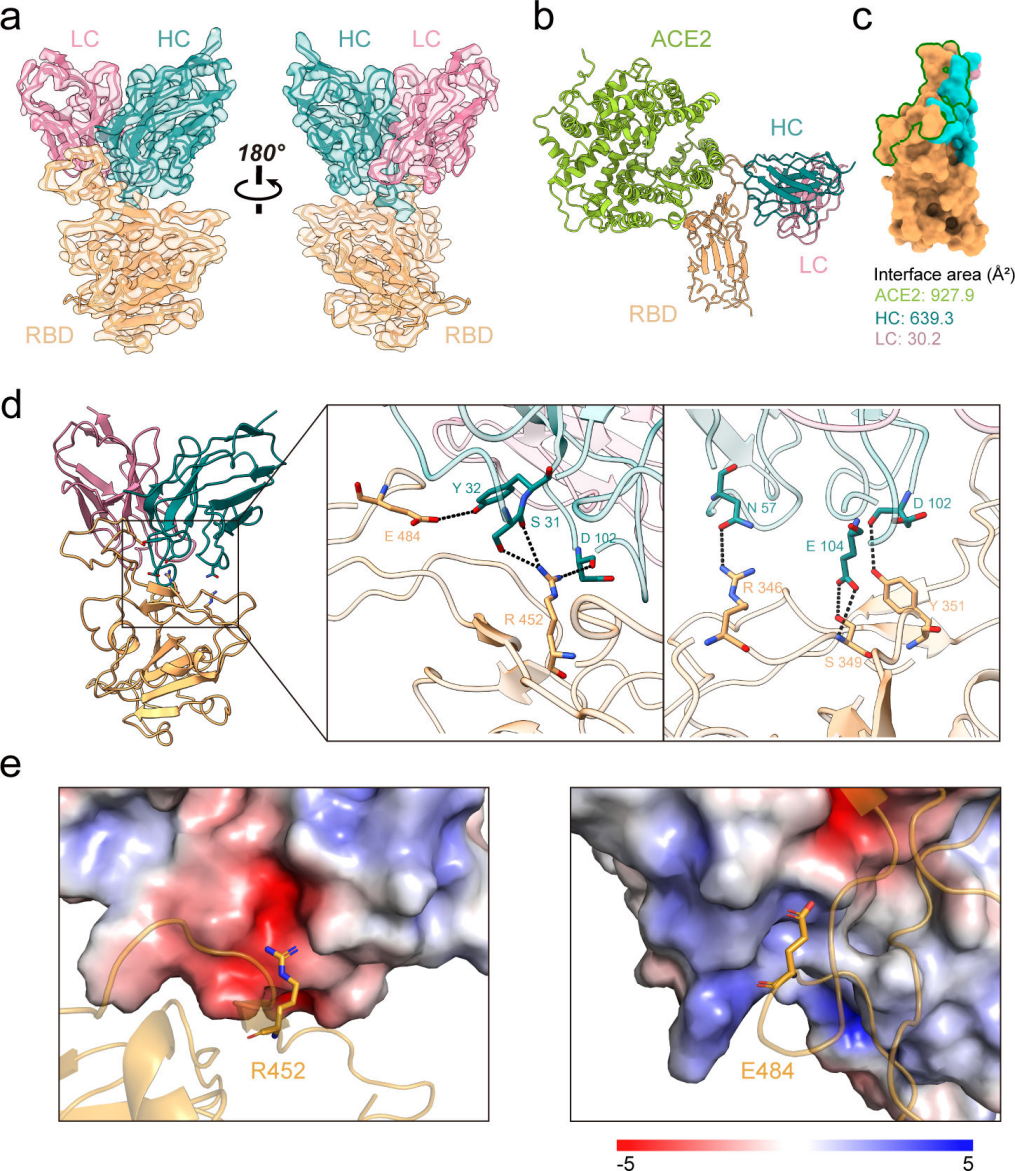
Structural basis of ConD-852 binding to Delta-RBD. (a) The structure of Delta-RBD and ConD-852 Fab complex. (b) Comparison between ConD-852 Fab and ACE2 (Protein Data Bank: 7W9I) binding to Delta-RBD. (c) The footprint of ConD-852 Fab and ACE2 on Delta-RBD. The epitopes of ConD-852 and ACE2 are colored cyan/pink and circled in dark green, respectively. (d) Interactions between ConD-852 and Delta-RBD. Delta-RBD is colored orange. ConD-852 is colored blue and pink for heavy chain and light chain, respectively. Amino acids are shown as sticks. Hydrogen bonds were shown as a black dashed line. (e) Electrostatic potential diagrams. R452 and E484 are shown as sticks. The electrostatic potential of ConD-852 is shown by the APBS electrostatic plugin of PyMOL. The electrostatic potential ranges from −5 (red) to +5 (blue) kT/e. Blue on the potential surfaces indicated a positive electrostatic potential, and red indicated a negative electrostatic potential, whereas the intermediate color indicated intermediate electrostatic potentials.

To elucidate the structural basis of ConD-852 recognizing the R452 residue, we determined the cryo-electron microscopy (EM) structure of ConD-852 Fab complexed with the Delta RBD. Fabs of P2C-1F11 (Class 1) and S304(42) (Class 4) were also included to improve the quality of complex formation. For clarity, the density maps and atomic models for P2C-1F11 and S304 were omitted (Fig. 4a; Fig. S2 and S3). We constructed an atomic model of the ConD-852 Fab-Delta RBD complex (Fig. 4b). The structure is determined at 3.28 Å resolution. The heavy and light chains of ConD-852 bury surface areas of 639.3 and 30.2 Å^2^ on the RBD, respectively (Fig. 4c). ConD-852 and ACE2 do not exhibit spatial clashes despite sharing some binding footprint. Five paratope residues of the ConD-852 heavy chain (S31, Y32, N57, D102, and E104) interact with five epitope residues on the Delta RBD (R346, S349, Y351, R452, and E484) (Fig. 4d). In contrast, the ConD-852 light chain almost does not participate in direct interactions with Delta RBD. The sequence analysis revealed that R346, S349, and Y351 were conserved in the WT, Alpha, Beta, Delta, Epsilon, Kappa, and Lambda virus strains (Fig. S4). Two non-conserved residues (R452 and E484) in the RBD possibly contribute to the neutralization activity of ConD-852 against different SARS-CoV-2 variants. R452 can form three potential hydrogen bonds with S31 and D102, and E484 can form one with Y32 of the ConD-852 heavy chain. Mutations of R452Q, R452L, E484K, or E484Q may cause loss of these hydrogen bonds. Additionally, R452 is in the middle of a negatively charged canyon formed by ConD-852 (Fig. 4e), which may explain the loss of neutralizing activity of ConD-852 against Lambda-carrying L452Q (Fig. 1e and 4e). In addition, E484 is present on the positive surface of ConD-852, which may explain the loss of neutralization of ConD-852 against Beta-carrying E484K (Fig. 1e and 4e).

### Impact of the L452R mutation on mAbs elicited by the WT SARS-CoV-2

**Fig 5:**
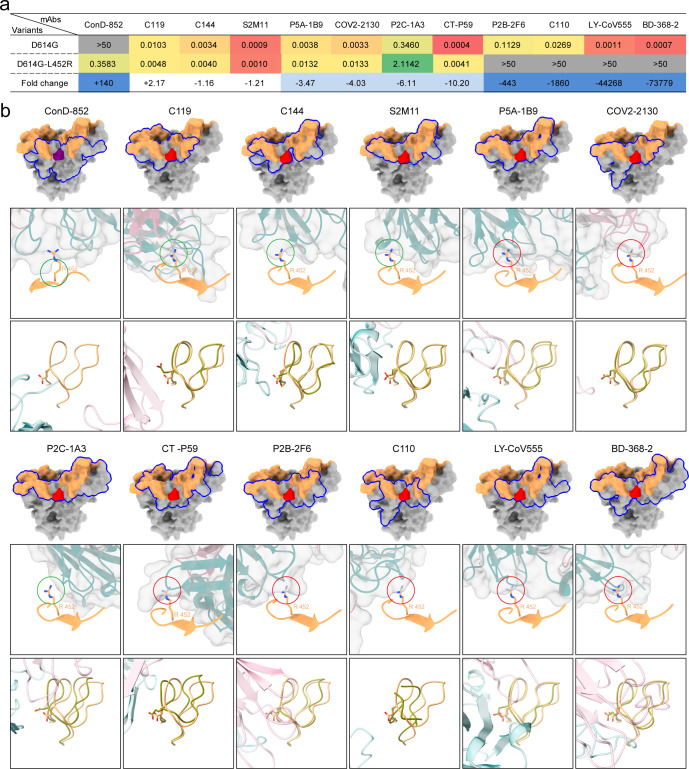
Neutralization of several monoclonal nAbs recognizing epitopes around position 452 against SARS-CoV-2 variants carrying L452 or R452. (a) The IC_50_ value and fold change of nAbs against SARS-CoV-2 D614G-L452 or R452 pseudoviruses. The data shown here are the means of two independent experiments. The IC_50_ values above 50 µg/mL are highlighted in gray. (b) (Top) RBDs are shown as surface, and footprints of different nAbs on RBD were circled out by blue lines. Epitope residues of ACE2 on RBD are colored orange. R452 is colored purple, and L452 is colored red. In the middle panel, atomic models of RBD-Fab (C119: 7K8W, C144: 7K90, S2M11: 7K43, P5A-1B9: 7CZX, COV2-2130: 7L7E, P2C-1A3: 7CDJ, CT-P59: 7CM4, P2B-2F6: 7BWJ, C110: 7K8V, LY-CoV555: 7 KMG, and BD-368–2: 7CHH) are aligned to Delta-RBD. R452 is shown as a stick. Regions where R452 was in contact with Fab are circled out. Green circles indicate that there was no clash, and red means a clash between R452 and the surface of Fab. (Bottom) Loops 470–491 are shown as cartoons; E484 is shown as a stick. Fab is shown as a cartoon and transparent surface. Heavy chains are colored teal, and light chains are colored pink.

To comprehensively investigate the impact of R452 and E484 on nAbs, further structure analysis was performed. According to the structure analysis (Fig. 4), ConD-852 belonged to the Class 2 antibody category. In our previous study (44), we summarized a total of 19 anti-RBD mAbs classified as Class 2. The binding footprints of these mAbs were analyzed using the PISA website (https://www.ebi.ac.uk/pdbe/pisa/). The footprints of 6 mAbs did not include residue 452, leaving 11 mAbs (C119, C114, S2M11, P5A-1B9, COV2-2130, P2C-1A3, CT-P59, P2B-2F6, C110, LY-CoV555, and BD-368–2) for further analysis. These structures were obtained from the Protein Data Bank and aligned to the Delta RBD-ConD-852 complex based on RBD. The neutralizing activities of these 11 mAbs against the WT_D614G_L452 and WT_D614G_R452 viruses were evaluated using a head-to-head comparison to assess their relative potencies (Fig. 5a). C119, C114, S2M11, P5A-1B9, COV2-2130, P2C-1A3, CT-P59, P2B-2F6, C110, LY-CoV555, and BD-368–2 were elicited by the infection or vaccination with the WT SARS-CoV-2, and hence, they could neutralize the WT_D614G_L452. The neutralizing activities of C119, C144, and S2M11 against WT_D614G_R452 were almost unaffected. The L452R mutation does not induce a steric hindrance effect with these three mAbs. By comparing the RBD in the Delta RBD-ConD-852 complex with those in WT RBD-C119, WT RBD-C144, and WT RBD-S2M11 complexes, it was observed that E484 in Loops 470–491 exhibits a slight movement (Fig. 5b; Fig.S5). Contrastingly, the neutralizing activities of P5A-1B9, COV2-2130, P2C-1A3, and CT-P59 against WT_D614G_R452 were reduced by 3.47- to 10.2-folds. P2B-2F6, C110, LY-CoV555, and BD-368–2 completely lost their neutralizing activities. The L452R mutation causes a steric-hindrance effect between R452 and these mAbs, except for P2C-1A3 (Fig. 5b). However, E484 of P2C-1A3 shifts at 2.3 Å, which is larger than that of C119, C144, and S2M11 (Fig. 5b; Fig. S5). The neutralizing activities of mAbs may be affected by both the steric hindrance of R452 and the shift of E484 on Loops 470–491.

### Strict restriction of ConD-852 binding to the R452 residue

**Fig 6:**
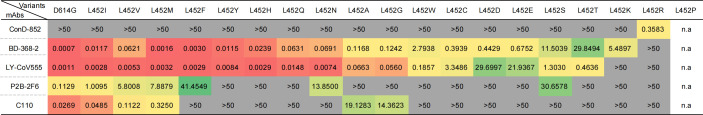
Neutralization of ConD-852 and L452-induced monoclonal nAbs against SARS-CoV-2 variants with single-residue substitutions at position 452. The IC_50_ values of ConD-852, BD-368–2, LY-CoV555, P2B-2F6, and C110 neutralizing against SARS-CoV-2 pseudoviruses with a series of single-residue mutations at 452. The data are means of three independent experiments.

Furthermore, we explored the broad neutralization of ConD-852 against 19 SARS-CoV-2 variants carrying a single mutation at the 452 position. As shown in Fig. 5, BD-368–2, LY-CoV555, P2B-2F6, and C110 completely lost their neutralizing activities against the L452R variant, suggesting that this residue had a substantial impact on recognition and neutralization. Thus, these four mAbs were selected as control. The L452P variant could not effectively infect the target cells and was not analyzed in this study. As shown in Fig. 6, Delta RBD-specific ConD-852 only neutralized the R452 variant, suggesting its strict restriction in the binding mode. Meanwhile, we also tested the neutralization of the above available four WT RBD-specific nAbs against this panel of single mutated pseudovirus at the 452 position. Despite some decline, these nAbs displayed some degree of cross-reactivity with L452 variants. Overall, these results indicated that ConD-852 induced by SARS-CoV-2 Delta infection was an R452-dependent monoclonal nAb and could not afford any mutation at the 452 position.

## DISCUSSION

Since the outbreak of the COVID-19 pandemic, researchers around the world have been working hard to discover potent vaccines and monoclonal nAbs to fight against the SARS-CoV-2 infection (37, 45–50). Broad-spectrum vaccines and nAbs have attracted increasing attention with the emergence of various SARS-CoV-2 variants such as Delta, Omicron BA.1, BA.2, BA.2.86, EG.5, JN.1, and KP.3 (52–55). Additionally, along with other researchers, we also focused on the efficacy of variant-specific vaccines and nAbs (15, 51–56). Lima et al. described the repertoire and epitope specificity of antibodies induced by primary exposure to the SARS-CoV-2 Beta or Gamma variant (57). Liu et al. and Reincke et al. separately cloned and identified several monoclonal nAbs from initially Beta-infected patients (27, 58). These two research groups demonstrated that Beta infection elicited cross-reactive nAbs and induced some variant-specific nAbs. Several Y501-dependent mAbs were isolated and studied in detail, which were derived from different patients and germline gene families but exhibited specific interactions with the Y501 residue. Moreover, the K417N and E484K mutations also elicited some potent mAbs, suggesting that these mutations in the RBD had altered the antigenic structure of the SARS-CoV-2 spike.

In this study, we isolated and characterized an R452-dependent anti-SARS-CoV-2 monoclonal nAb, ConD-852, from a COVID-19 convalescent patient primarily infected with the Delta variant. The heavy chain of ConD-852 utilized the IGHV3-33 germline gene with 16 amino acids of the complementarity-determining region 3 (CDR3) and 0.69% of somatic hypermutations. The light chain of ConD-852 was derived from the IGKV3-20 with 10 amino acids of CDR3 and no genetic mutations. Isolation of ConD-852 revealed that the mutated R452 residue was immunogenic and could induce a potent neutralizing antibody response, similar to K417N, E484K, and N501Y (27, 57, 58). Cryo-EM structural analysis of ConD-852 complexed with Delta RBD revealed its close interactions with R346, S349, Y351, R452, and E484. Although ConD-852 forms a few hydrogen bonds with Delta RBD, it maintains a high binding affinity at the nanomolar level. Combined with the results of binding epitope identification, ConD-852 was a kind of Class 2 nAb. Greaney et al. also reported that the primary infection of the Delta variant mainly induced an antibody response by binding to Class 1 and Class 2 antibody epitopes, which were strongly affected by mutations appearing at site K478 (59). However, ConD-852 identified in this study mainly recognized the region around R452 on the Delta RBD. Saturation mutagenesis at the 452 position on the RBD confirmed that ConD-852 restricted mAb binding to the R452 residue, and further mutations at the 452 position may totally abolish its neutralizing activity.

**Fig 7:**
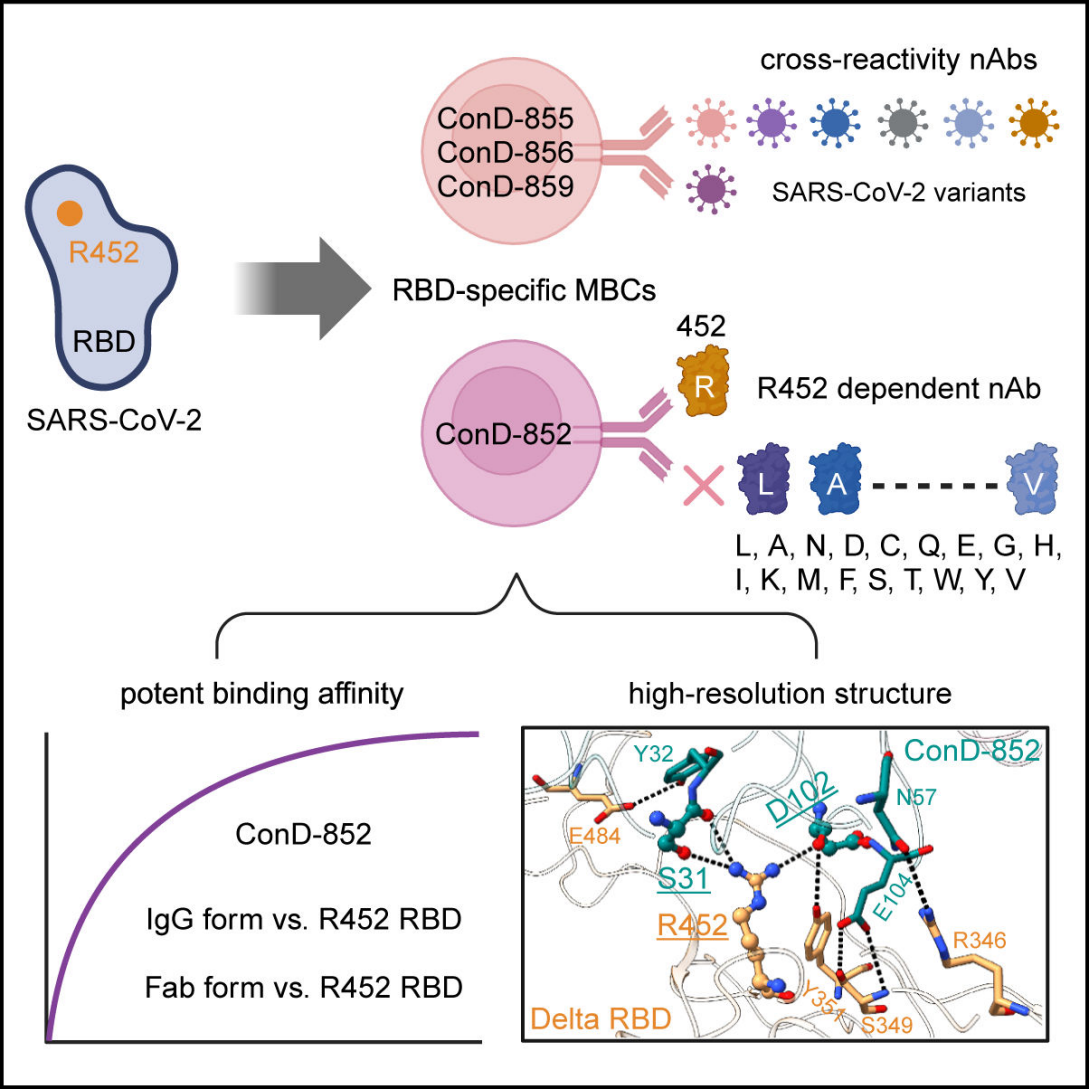
Overall summary of the research findings in this study. The mutated R452 residue still has immunogenicity and induces an R452-dependent monoclonal nAb named ConD-852. Cryo-EM structure reveals the mechanism of ConD-852 binding to the R452 yet not binding to any other amino acid at position 452.

Overall, as shown in Fig. 7, we identified an R452-dependent monoclonal nAb from a Delta-infected donor and confirmed the immunogenicity of the R452 residue, indicating that other variants or vaccines carrying the L452R mutation could also induce potent nAbs in humans. Moreover, we reported a high-resolution structure for ConD-852 binding to epitopes around the R452 residue, explaining its strict restriction and expanding our understanding of antigen-antibody interactions. More R452-dependent mAbs need to be identified from SARS-CoV-2 variant-infected patients and should be systematically studied in the future.

## MATERIALS AND METHODS

### Biological samples

Six patients with confirmed infection with the SARS-CoV-2 Delta variant were included in this study. The mean age was 23 years, and two patients were female. Among these patients, none received vaccine and antibody therapy. The participants had provided written informed consent for sample collection and subsequent analysis. All plasma and peripheral blood mononuclear cell (PBMC) samples were stored at the BioBank of Shenzhen Third People’s Hospital. All plasma samples were stored at −80°C and heat-inactivated at 56°C for 1 h before use. PBMCs were maintained in freezing medium and stored in liquid nitrogen before use.

### Pseudovirus neutralization assay

Pseudoviruses were generated via the co-transfection of HEK-293T cells (American Type Culture Collection) with human immunodeficiency virus backbones expressing firefly luciferase (pNL4-3.Luc.R-E-) and pcDNA3.1 (Invitrogen) expression vectors encoding the respective spike proteins. After 48 h of transfection, cell culture supernatants containing the virus were collected through centrifugation. The neutralization activities of the test samples (plasma or monoclonal antibody) were evaluated by incubating the serially diluted samples with an equal volume of pseudovirus at 37°C for 1 h. The virus-sample mixture was then transferred to an all-white 96-well cell culture plate; subsequently, HEK-293T-hACE2 cells were added to the plates. The culture medium was removed 48 h post-incubation, and 100 µL of the Bright-Lite Luciferase reagent (Vazyme Biotech) was added to the plates. After a 2 min shock incubation at room temperature (RT), the cell plates were measured by luminescence using the Varioskan LUX multimode microplate reader (Thermo Fisher Scientific). Half-maximal inhibitory dilution (ID_50_) and half-maximal inhibitory concentration (IC_50_) were calculated using GraphPad Prism version 8.0 software by log (inhibitor) vs normalized response − variable slope (four parameters) model (60).

### Isolation of monoclonal antibody from a SARS-CoV-2 Delta-infected donor

Thawed PBMCs were stained with an antibody cocktail containing CD19-PE-Cy7, CD3-Pacific Blue, CD8-Pacific Blue, CD14-Pacific Blue, CD27-APC-H7, IgG-FITC (BD Biosciences), and IgA-FITC (Miltenyi Biotec) to detect IgG+ or IgA+ memory B cells. SARS-CoV-2 Delta-S1 with a C-His-biotin tag (Sino Biological) was used as a probe to select antigen-specific single cells. To exclude non-specific staining, two streptavidin secondary antibodies labeled with APC and PE (BD Biosciences) were used to recognize the Delta-S1 bait. Flow cytometric data were acquired using a BD Aria II (BD Biosciences) and analyzed using FlowJo software (TreeStar). Single B cells were sorted in 96-well PCR plates containing lysis buffer, followed by RT-PCR and nested PCR to amplify the variable regions of the heavy and light chains (37). Variable genes were sequenced by Sangon Biotech, synthesized by GenScript, and separately cloned into full-length IgG1 heavy and light chain expression vectors. Monoclonal antibodies were expressed by co-transfection of 293 F cells with paired heavy- and light-chain plasmids and purified from the culture supernatants using a protein A column (GenScript).

### ELISA

SARS-CoV-2 Delta-S1, Delta-RBD, and WT-RBD proteins (Sino Biological) were coated onto 96-well plates at 4°C overnight. The plates were washed with phosphate-buffered saline with Tween 20 (PBST) buffer and blocked with 5% skim milk and 2% bovine albumin in phosphate-buffered saline (PBS) at RT for 1 h. Diluted mAbs were added to the wells and incubated at 37°C for 1 h. Next, the plates were washed, and horseradish peroxidase (HRP)-conjugated goat anti-human IgG antibodies (ZSGB-BIO) were added and then incubated at 37°C for 1 h. The samples were incubated with TMB substrate (Sangon Biotech) at RT for 20 min, and the reaction was stopped using 2 M H_2_SO_4_. The readout was detected at a wavelength of 450 nm (61).

### Competition ELISA

The 96-well plates were coated overnight with SARS-CoV-2 Delta-RBD or Delta-spike protein (Sino Biological) at 4°C. The plates were washed with PBST buffer and blocked with 5% skim milk and 2% bovine albumin in PBS at RT for 1 h. mAbs were serially diluted (threefold) and mixed with an equal volume of HRP (Abcam) conjugated ConD-852; the mixture was added to the plates and incubated at 37°C for 1 h. Subsequently, the TMB substrate (Sangon Biotech) was added and incubated at RT for 20 min, and the reaction was stopped by 2 M H_2_SO_4_. The readout was detected at a wavelength of 450 nm. The competitive effect was determined by comparing the ratio of different mAbs to the negative control (28, 34, 52, 62).

### Binding affinity analysis by SPR

The binding affinities of mAbs to the SARS-CoV-2 Delta-S1 protein and SARS-CoV-2 Spike RBD-His tag (L452R) (Sino Biological) were assessed using the Biacore 8 K system (GE Healthcare). Specifically, one flow cell of the CM5 sensor chips was covalently coated using Delta-S1 or WT RBD (L452R) in 10 mM sodium acetate buffer (pH 5.0) to obtain a final response unit of approximately 250, whereas the other flow cell was left uncoated and blocked as a control. All the assays were run at a flow rate of 30 µL/min in HBS-EP buffer (10 mM HEPES, pH 7.4, 150 mM NaCl, 3 mM EDTA, and 0.05% Tween 20). Serially diluted antibodies were injected for 60 s, respectively, and the resulting data were fitted to a 1:1 binding model using the Biacore Evaluation software (GE Healthcare)(63). Each measurement was performed two times, and individual values were used to produce the mean affinity constant.

### Cryo-EM sample preparation and data collection

RBD of SARS-CoV-2 Delta spike protein was incubated with Fabs of ConD-852, P2C-1F11, and S304 for 30 min on ice at a mole ratio of 1.0:1.5:1.5:1.5. The sample was loaded onto a Superdex 200 Increase 10/300 Gl column for sample separation. The fractions containing the complex were pooled and concentrated to about 0.5 mg/mL and immediately used for cryo-EM grid preparation with a Vitrobot Mark IV (FEI) at 4°C and 100% humidity. After waiting for 2.5 s, the grid was blotted for 4 s with a blot force of 0 and immediately plunged into liquid ethane. Grids were transferred into the Titan Krios transmission electron microscope and collected with SerialEM software. Movies were collected with a slit width of 20 eV and a defocus range from −1.5 to −2.5 µm, with a pixel size of 0.66 Å/pixel using a K3 camera. Exposures were performed with a total dose of 50 e^−^/Å^2^, which were fractionated into 32 frames (64, 65).

### Cryo-EM data processing and model building

A total of 3,523 movies were collected, containing 2,466 tilt 0° and 1,057 tilt 40°. Movies were corrected for beam-induced drift using MotionCor2. The defocus values were estimated with Gctf. Micrographs were imported into cryoSPARC to pick particles. Particles were picked out by a Blob picker. After several rounds of 2D classification, 255,061 good particles were selected for ab initio reconstruction. Hetero refinement and non-uniform refinement were executed. Finally, 116,698 particles were used for non-uniform refinement, acquiring a density map at a resolution of 3.28 Å. The predicted RBD of the Delta spike protein and ConD-852 by AlphaFold2 were used as the initial model and fitted into the density map by using Chimera. The atomic model of the RBD/ConD-852 complex was further manually adjusted in Coot and refined in Phenix through the real-space refinement program. The refinement cycle was repeated, and the quality of the final 3D atomic model was evaluated using MolProbity (64, 65). Refer to Fig.S2 and Table S2 for details of data collection and processing. The structure figures were prepared using PyMOL (http://www.pymol.org) or ChimeraX.

## Data Availability

The structure coordinates for the Delta-RBD-ConD-852 complex have been deposited in the Protein Data Bank under accession code 9L6C. The corresponding cryo-EM density maps have been deposited in the Electron Microscopy Data Bank under accession number EMD-62852. This paper does not report original code. Any additional information required to reanalyze the data reported in this paper is available from the lead contact upon request.

## SUPPLEMENTAL MATERIAL

Supplemental material jvi.01844-24-s0001.pdf Figures S1 to S5; Table S1.

ASM does not own the copyrights to Supplemental Material that may be linked to, or accessed through, an article. The authors have granted ASM a non-exclusive, world-wide license to publish the Supplemental Material files. Please contact the corresponding author directly for reuse.
