# Supplementary material for: Structure and function of an unusual R452-dependent monoclonal antibody against SARS-CoV-2
Source: J Virol. 2025 Apr 8;99(5):e01844-24. doi: 10.1128/jvi.01844-24 (PMC12090765; doi:10.1128/jvi.01844-24)
Supplement: Supplemental material — Figures S1 to S5; Table S1. [file jvi.01844-24-s0001.pdf]

Supplementary information

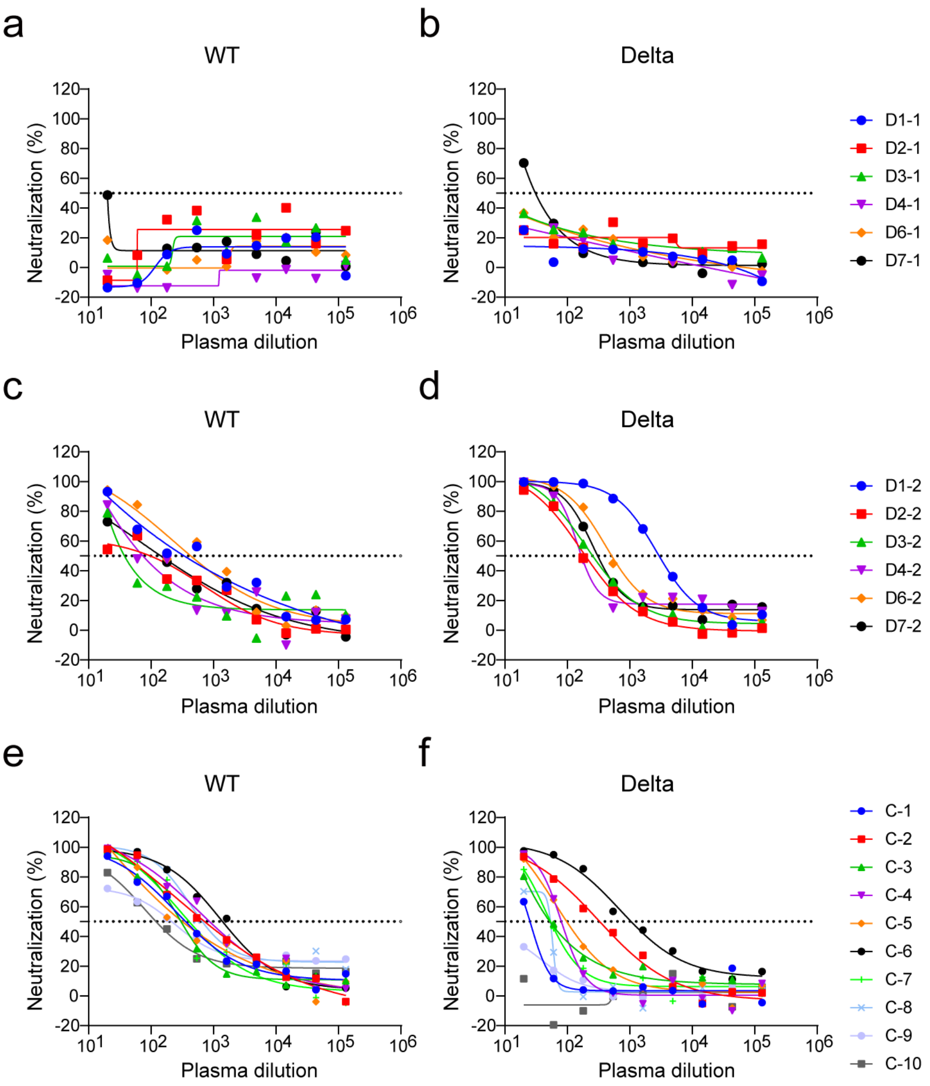

Figure S1. Neutralization of plasma samples against SARS-CoV-2 pseudoviruses of WT and Delta.

(a and b) The neutralization of WT and Delta pseudoviruses by plasma samples from 6 patients infected with Delta variant at admission.

(c and d) The neutralization of WT and Delta pseudoviruses by plasma samples from 6 patients infected with Delta variant at convalescent.

(e and f) The neutralization of WT and Delta pseudoviruses by plasma samples from 10 SARS-CoV-2 WT convalescent individuals.

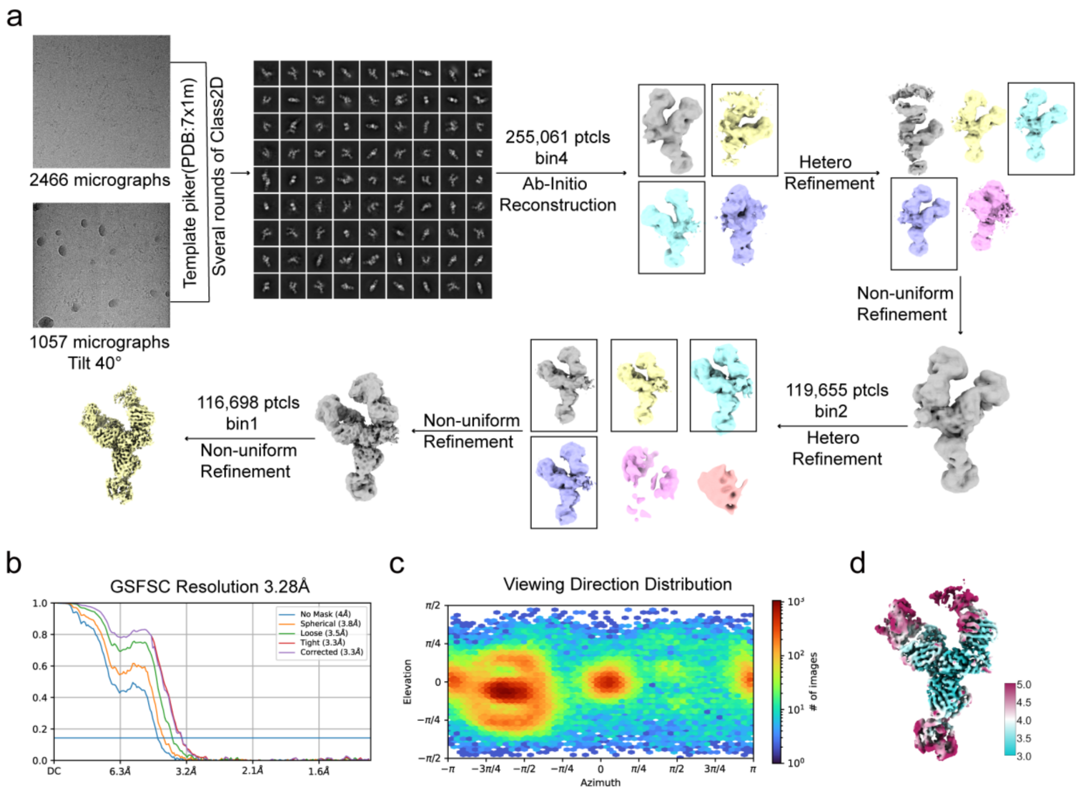

Figure S2. Cryo-EM image-processing workflow for Delta-RBD, ConD-852, P2C-1F11, and S304 complex.

(a) Representative cryo-EM micrographs, results of 2D classification, initial model, hetero refinement and the final density map. **(b)** FSC curve, **(c)** Viewing Direction Distribution and **(d)** local resolution for the Delta-RBD and Fabs complex.

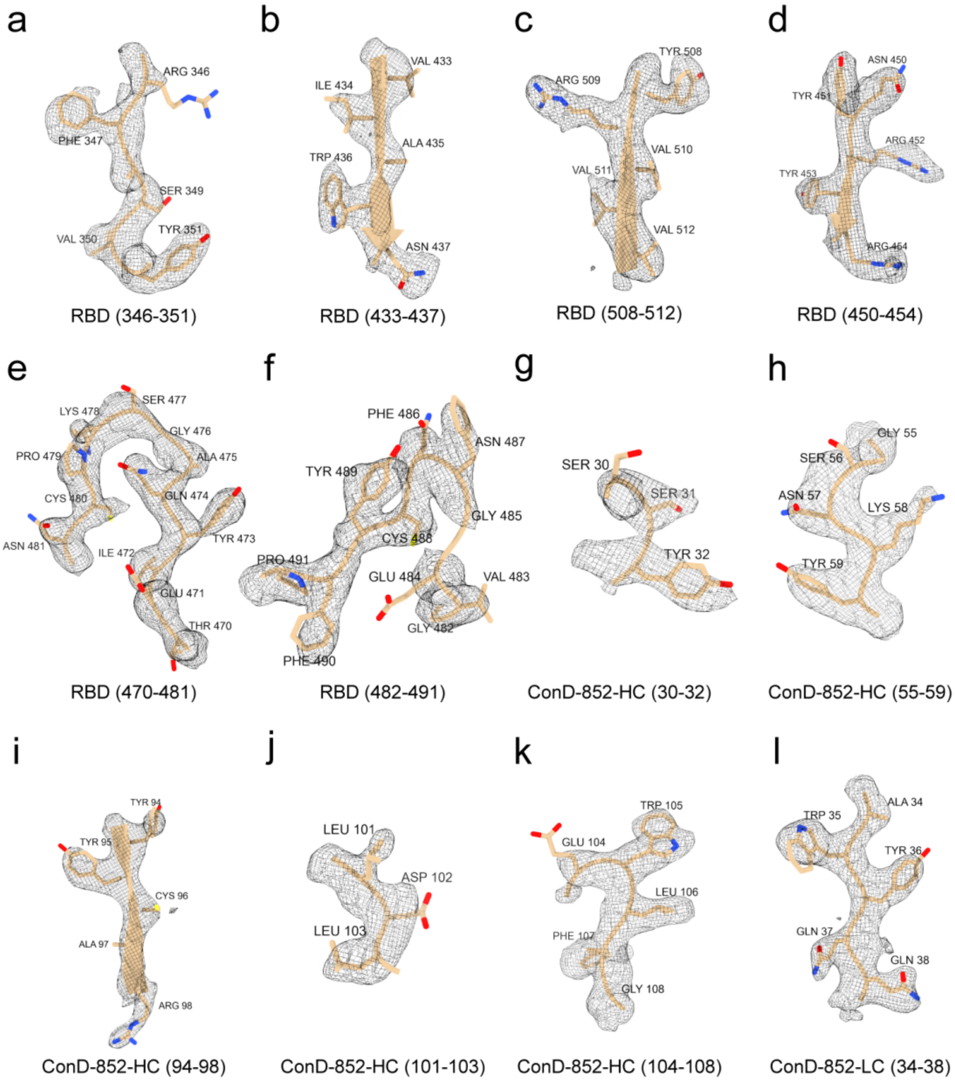

Figure S3. Cryo-EM density maps for Delta-RBD and ConD-852 complex. **(a-f)** Local density maps and models of Delta RBD. **(g-k)** Local density maps and models of ConD-852 heavy chain. **(l)** Local density maps and models of ConD-852 light chain.

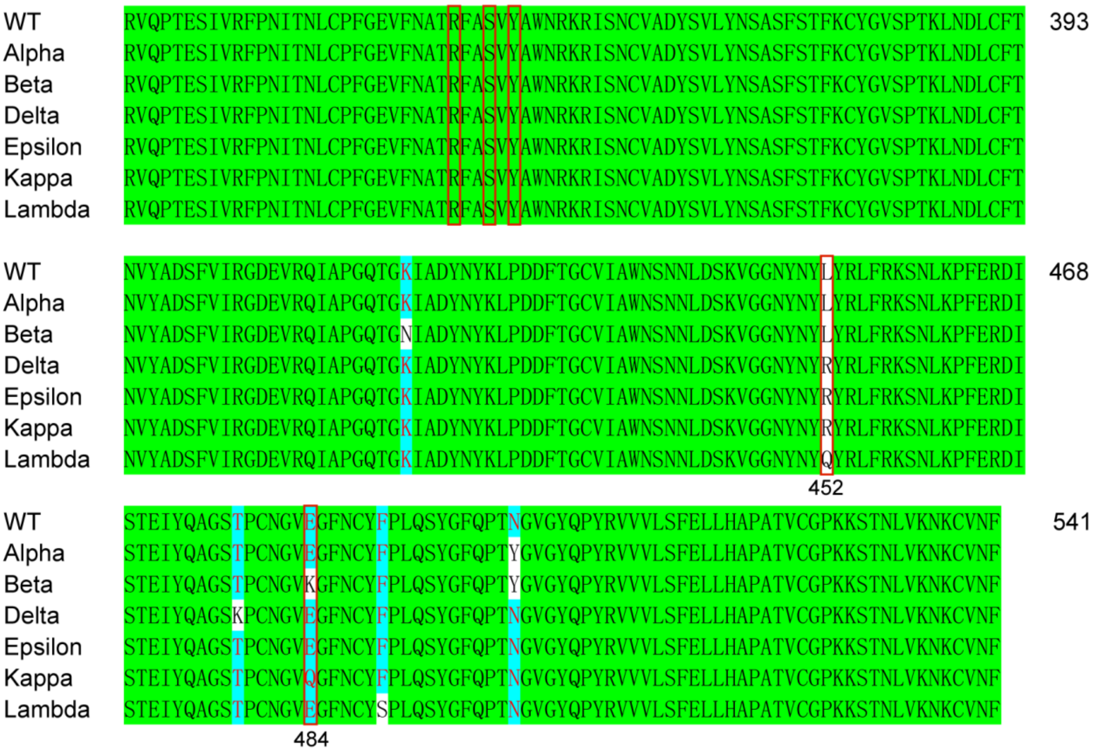

Figure S4. Sequence alignment of RBD proteins among WT, Alpha, Beta, Delta, Epsilon, Kappa, and Lambda.

The key interaction residues were boxed out by red boxes. Sites 452 and 484 were marked.

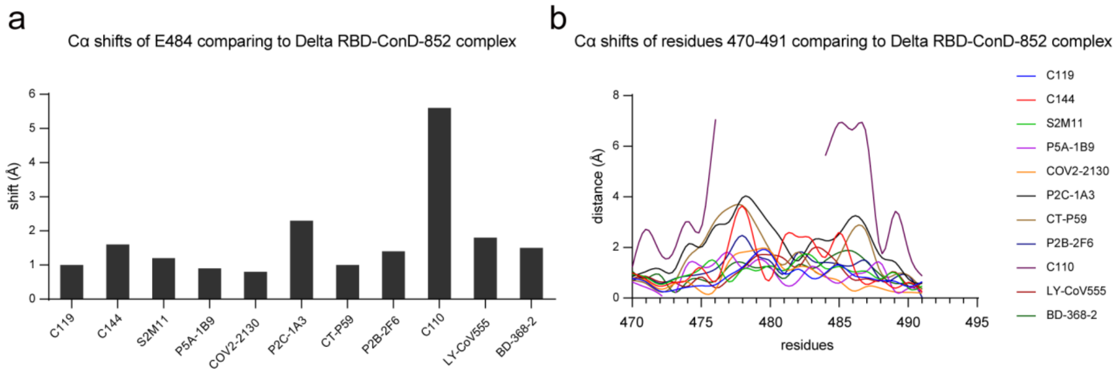

Figure S5. Cα shifts of (a) E484 and (b) residues 470-491 comparing to Delta RBD-ConD-852 complex.

Table S1. Cryo-EM data collection and processing, model building and refinement statistics.

Data Collection & Image Processing	
Microscope	FEI Titan Krios
Magnification	105K
Voltage(kV)	300
Automation software	SerialEM
Total dose (e–/Å2)	50
Frames	32
Defocus range (μm)	-1.5-2.5
Pixel size (Å/pixel)	0.66
Symmetry imposed	C1
Micrographs used (no.)	3523
Final particles images	116,698
Overall resolution(Å)	3.28
Model Building & Refinement	
Composition	
Nonhydrogen atoms	6766
Protein residues	874
Ligands	0
Bonds (RMSD)	
Length (Å) (# > 4s)	0.002(0)
Angles (°) (# > 4s)	0.567(1)
MolProbity score	1.77
CaBLAM outliers (%)	4.14
Clashscore	6.86
Rotamer outliers (%)	0.27
C-beta outliers (%)	0.0
Ramachandran plot	
Favored (%)	94.19
Allowed(%)	5.47
Outliers(%)	0.35
